# Diné teachings and public health students informing peers and relatives about vaccine education: Providing Diné (Navajo)-centered COVID-19 education materials using student health messengers

**DOI:** 10.3389/fpubh.2022.1046634

**Published:** 2022-12-14

**Authors:** Marissa Tutt, Chassity Begay, Shawndeena George, Christopher Dickerson, Carmella Kahn, Mark Bauer, Nicolette Teufel-Shone

**Affiliations:** ^1^Center for Health Equity Research, Northern Arizona University, Flagstaff, AZ, United States; ^2^Diné College, School of Science, Technology, Engineering and Math, Shiprock, NM, United States

**Keywords:** COVID-19 education, vaccine hesitancy, vaccine education, health messengers, Navajo

## Abstract

**Introduction/background:**

On 9 April 2021, the Centers for Disease Control and Prevention (CDC) reported that only 19. 9% of United States (US) adults were fully vaccinated against COVID-19. In that same week, the Navajo Nation (NN) reported that 37.4% of residents were fully vaccinated, making the NN a leader in the uptake of COVID-19 vaccines. Despite high vaccination rates, vaccine hesitancy exists within the NN. The Diné (Navajo) Teachings and Public Health Students Informing Peers and Relatives about Vaccine Education (RAVE) intervention was designed to utilize trusted health messengers as an effective means to address adults' vaccine concerns and hesitancy.

**Methods:**

The research team used COVID-19 vaccine materials developed in a previous collaboration with non-Navajo tribal communities and publicly available materials. Diné Traditional Knowledge Holders (TKHs) were interviewed to develop and incorporate Diné-specific information on individual and collective health behaviors into the RAVE materials. These drafted health education materials were presented to NN community health representatives (CHRs) and Diné public health students using a consensus panel approach. NN residents who participated in the intervention completed a 16-element retrospective pretest.

**Results:**

The adaptation and tailoring process of materials yielded 4 health education materials. The students recruited 46 adults for health education sessions. These participants then completed the retrospective pretest. Changes in the 16 elements were in the desired direction, although only six were significant: four related to attitudes and two concerned with vaccination intention. Participants were more likely to consider vaccination and to try to get vaccinated after the education session.

**Discussion:**

Trusted messengers and culturally centered materials have been identified as effective means of health behavior education with Native American audiences. RAVE applied these intervention elements by (1) training Diné College public health students to leverage their cultural knowledge and social relationships (cultural and social capital) to recruit vaccine-hesitant adults and provide education; (2) building on previous understanding of Native American communities' vaccine concerns; and (3) integrating Diné perspectives on individual and collective health into the adaptation of materials designed for general audiences; this knowledge was gained from interviews with TKHs.

## Introduction

American Indian/Alaskan Native (AI/AN) populations have been negatively impacted by the COVID-19 pandemic due to racial inequity, historical trauma, and health disparities resulting in an incidence of positive COVID-19 cases 3.5 times that of non-Hispanic whites (1). The Navajo Nation (NN) has the largest tribal enrollment at 332,129 and is the largest Native American reservation in the United States (US). The NN spans parts of Arizona, New Mexico, and Utah, where over 173,000 enrolled Diné citizens reside (2, 3). In May 2020, the NN surpassed both New York and New Jersey for the highest per-capita COVID-19 infection rate in the US with 2,304 positive cases per 100,000 residents, while the overall US rate was 636 positive cases per 100,000 residents (4). The increase in COVID-19 cases, and ultimately the highest rate of COVID-19-related mortality, is attributed to NN residents living in multigenerational homes, having limited access to running water and resources, and lacking social trust in external social systems (5–7). Although more NN residents were fully vaccinated at 37.4%, compared to 19.9% in the US adult population at the same time period, vaccine hesitancy was still evident, preventing some NN residents from receiving the vaccine (5, 8, 9). Vaccine uptake is critical in Native American populations as national data indicate that Native Americans have disproportionally high levels of pre-existing health conditions and have the highest rates of COVID-19-related mortality compared to other US populations (10, 11). Prior to the COVID-19 pandemic, Native Americans had higher vaccine rates than the general US populations, noted particularly for influenza and human papillomavirus infection (12, 13). Based on a review of social media discussions among NN residents, COVID-19 vaccine hesitancy is grounded in historical mistrust of the government (14, 15). This article describes the development of materials and the outcome of the vaccine safety education sessions developed for NN residents.

The Diné Teachings and Public Health Students Informing Peers and Relatives about Vaccine Education (RAVE) intervention were designed to integrate trust and culture to address adults' vaccine concerns and hesitancy. RAVE's objective was to increase NN adult residents' knowledge of the COVID-19 vaccines to encourage vaccination uptake. Undergraduate public health students at Diné College, a tribal college located on the NN, were identified as trusted messengers who could be trained to deliver and provide culturally centered, scientifically accurate vaccine-safety information to NN residents. These trusted health messengers used Diné-specific relationality and etiquette to talk to their peers and relatives who were hesitant about vaccination.

In addition to the Diné College public health students, RAVE engaged two NN community health representatives (CHRs) in the intervention development. CHRs, community health workers (CHWs), and lay health educators are a well-recognized workforce that helps reduce health disparities and improve health equity among underserved populations through direct home-based care and education (16). In NN, CHRs play a vital role as cultural mediators and are frontline public health workers, trusted by their communities. CHRs, CHWs, and lay health educators' duties were put on hold due to the COVID-19 pandemic and transitioned to crisis management focused on disaster response (17, 18).

Once trained in a classroom setting, Diné College public health students were able to deliver vaccine education to their peers and family members relying on their social and cultural obligation through *K'é*, a core cultural teaching referring to descent, clanship, and kinship, to inform and contribute to the relevance of necessary information (19, 20).

## Methods

### Developing education materials

The intervention team drew on the Theory of Planned Behavior (TPB) (21), COVID-19 vaccine education materials developed in a previous collaboration with non-Navajo tribal communities, and the Arizona Community Health Workers (AzCHOW) Association (22), as well as traditional Diné concepts of individual and collective health. The TPB posits that three core components, specifically attitudes, subjective norms, and perceived behavioral control, link beliefs to behaviors and thus shape an individual's behavioral intentions. Four Traditional Knowledge Holders (TKHs) were interviewed to understand COVID-19 and how vaccines fit into Diné views of maintaining wellness. The Navajo investigators on the research team reviewed the interview transcripts, identified the key concepts, and integrated the TKHs' Diné perspectives on individual and collective health into the adaptation of materials designed for RAVE's audiences. The in-depth analysis of the TKH interviews will be discussed in a forthcoming manuscript. Further adaptations were informed by health education materials from the Johns Hopkins Center for American Indian Health and the Navajo Department of Health (NDOH). The research team began developing the COVID-19 education materials using a free-to-use, online graphic-design tool called Canva^tm^.

### Consensus panels

The research team engaged in a consensus-based, decision-making method to review and modify vaccine education materials with CHRs and student health messengers. Consensus-based decision-making, or a consensus panel, involves the group to actively participate in making a decision or a plan in which all members are comfortable (23). This approach was used to leverage the collective knowledge of the CHRs and Diné College public health students to contribute to the comprehension and appeal of the COVID-19 education materials. Key foci of materials were to ensure the approach was culturally appropriate and addressed knowledge gaps about the COVID-19 vaccines, for example, the traditional perspectives of the pandemic and the difference between quarantine and isolation. The questions used in the consensus panels were based on criteria for language and content, format and organization, and imagery and colors.

The draft COVID-19-education materials were presented to CHRs in the first consensus panel. After the first consensus panel feedback, the research team began revising the materials. Once materials were complete, a second consensus panel was conducted with Diné College public health students to further refine the materials. Once the CHRs and public health students approved the final revisions and provided any final remarks, the materials were saved as final electronic PDF files or printed.

### Health messenger training

A total of 16 Diné College students were enrolled in a special topics course and were trained using the aforementioned culturally centered materials to become health messengers providing vaccine safety education. Based on the aforementioned work in non-Navajo tribal communities, Native Americans described being motivated to get vaccinated to keep their families healthy; this commitment to family wellbeing was incorporated into the vaccine safety materials. In addition, students were provided with extensive information on the technology of vaccine development, clinical trials, and vaccine myths propagating on social media.

Students gained confidence in delivering materials through motivational interviewing (24). Once student health messengers were trained, they were tasked to recruit between 5 and 10 peers and/or relatives to whom they would deliver and discuss the vaccine-education materials. Eligibility for recruitment included individuals who have not received the COVID-19 vaccine, aged 18 years and older, have been identified as any race and gender, and have resided or worked on the NN. Once student health messengers identified their potential participants, they scheduled a one-on-one or group session either in-person or virtually through Zoom^tm^. At the beginning of each session, health messengers read and answered questions related to the Human Subject consent form and secured informed consent for participation *via* electronic or hard copy signatures.

### Retrospective pretest

To determine the effectiveness of the intervention, student health messengers administered a retrospective pretest to participants receiving the health message. The questionnaire asked participants to report their attitudes, perceived behavioral control, subjective norms, and intent to receive a COVID-19 vaccine after the education session, and then report their views before the session through the retrospective pretest. Data were collated in Microsoft Excel and chi-square tests were performed using OpenEpi.com (25) which makes basic data analysis available *via* the Internet to users who might otherwise not have access to statistical software. After each student health messenger session, the retrospective pretest was administered *via* hard copy or electronically through SurveyMonkey^tm^. As guided by the TPB, the retrospective pretest (26) sought to evaluate shifts in attitudes and intention to get vaccinated as well as influences, such as perceived behavioral controls and subjective norms. The retrospective pretest was used in place of a traditional pretest/posttest to reduce subjects' burden as only one administration is required and minimizes bias since participants often overestimate their knowledge of a topic in a classic pretest/posttest design (26).

## Results

### Consensus panel

Two CHRs and 13 public health students participated in the material development consensus panel, of which over 75% of consensus panel members identified as Diné and women. A total of three 1-h-long consensus panels were conducted with the CHRs and student health messengers, 1 for the CHRs, and 2 for the student health messengers. CHRs and student health messengers provided feedback on the health education materials in the following areas: language and content, format and organization, and images and colors. The feedback provided include clarifying wording or using less scientific terms, adding borders for better organization, making images Navajo-specific, and/or validating the information on the education materials. Additional feedback can be seen in Supplementary Table 1 (consensus panel results).

### Final health education materials

A total of four education materials were created as follows: COVID-19 Vaccines, COVID-19 FAQs and Myths, Quarantine vs. Isolation, and Traditional Knowledge of COVID-19. The final version of the education materials can be seen in Supplementary Table 2 (COVID-19 health education materials).

### Evaluation of participant education

A total of 46 individuals completed the retrospective pretest to assess changes in attitudes, perceived behavioral control, subjective norms, and intent to receive the vaccine. Possible answers used either a binary scale, “A Great Deal/Not at All,” or one of the two four-point, Likert-type scales: “Strongly Agree” to “Strongly Disagree” or “Very Likely” to “Very Unlikely.” Questions with four possible answers were collapsed into the binary outcome for analysis. No participant demographics were collected and not all participants answered every question, so total responses ranged from 43 to 46. All responses indicated a change in the desired direction, including five questions where the desired direction would be a negative change from pretest to posttest.

Four of the nine attitude questions and two of the three intent questions demonstrated a statistically significant change. The single perceived-behavioral-control and three subjective-norm questions were approached but they did not attain significance. Statistically significant changes occurred with increases in the number of participants who believed that getting the COVID-19 vaccine was a good idea (56.5%), that the vaccine would prevent COVID-19 (66.3%), and that the vaccines would protect the community (53.3%); fewer believed that the research conducted on the vaccines was insufficient (−22.5%). Willingness to consider getting the COVID-19 vaccine (42.9%) and intent to get the vaccine (77.3%) both significantly increased, demonstrating that using students trained as health messengers is effective in changing attitudes and intents surrounding vaccination status in individuals with which they are familiar (refer to Supplementary Table 3). The questionnaire asked participants to report their change in attitudes, perceived behavior control, subjective norms, and intent to receive the vaccine by health messaging recipients.

## Discussion

The RAVE intervention illustrated that undergraduate public health students who have social and cultural capital in a community can be effective health messengers. Social capital refers to the non-financial resources available through social networks, most notably support gained through the interpersonal connections and norms of trust and reciprocity on which networks depend (27). Cultural capital is obtained from resources based on shared values, behavioral norms, and culture-specific knowledge acquired by group learning occurring through shared experiences and histories as well as cultural knowledge, stories, and activities (28). This relational asset has received limited attention in health promotion literature but has been discussed in reference to nursing education as a means to enhance the quality of care and engage older adults in volunteerism (28–30). RAVE demonstrates these assets can contribute to initiating an atmosphere of trust, credibility, and caring. *K'é* embodies social and cultural capital within the Diné relational environment. A core element of *K'é* is honoring individual and familial relationships to people and ultimately all living things, thus guiding positive behaviors and interactions of Diné people with relatives (31, 32). *K'é* supported Diné public health students' ability to establish an earnest social connection, allowing them to recruit individuals who were distrustful of the healthcare system and gain consent from these individuals to hear about vaccine safety. As the information was being delivered by a “relative,” the health message was accepted as credible and the intention of the messenger was interpreted as sincere, grounded in the messenger's legitimate concern for the participant's health (31, 32).

Although *K'é* is a distinctively Diné concept, RAVE demonstrates that significant attitudinal and intentional shifts in health behaviors can occur when both the message and the messenger share social and cultural capital with the recipient. Health promotion efforts have documented that trust and empathy are enhanced with the ethnic concordance of providers and patients (33) and the ethnic and socio-economic concordance of community health workers and community members (34). RAVE demonstrated that trust and empathy can be enhanced when the message is culturally centered and shaped by the messengers themselves. Although not directly documented in RAVE's evaluation, participants may have been particularly receptive to these predominantly young adults aspiring to contribute to the public health workforce of the NN. Indigenous people have long identified future generations as the path to build tribal capacity and self-determination (35). Using an academic-course setting can be effective in training students, the majority of whom were members of the community, as trusted messengers to deliver health education to peers and family. By doing so, attitudes and intentions toward the uptake of health practices can be changed in targeted populations who might otherwise be resistant to those practices. RAVE suggests that undergraduate students are an underutilized public health resource in Indigenous communities and perhaps in other underserved communities striving toward health equity.

### Limitations/strengths

The limitations of RAVE were the small number of student participants and the lack of peers and family members who were recruited. At least 20 students were initially enrolled in the course, but a few withdrew because they had other time commitments or felt it would be too challenging to recruit participants. Of the 16 students who remained in the class, only 12 were able to recruit peers and family members because many of the individuals they knew had already been vaccinated. The strength of RAVE was the long-term partnership between Diné College and Northern Arizona University through the NIH-NIGMS supported mechanism, the Navajo Native American Research Center for Health (NARCH) Partnership. The senior and junior investigators, staff, and students of the Navajo NARCH bring a diversity of experience, skills, and passion to design, implement, and evaluate innovative strategies to achieve health equity for the Diné people.

## Data availability statement

The original contributions presented in the study are included in the article/Supplementary material, further inquiries can be directed to the corresponding author.

## Ethics statement

The studies involving human participants were reviewed and approved by Northern Arizona University Institutional Review Board, Diné College Institutional Review Board, Navajo Nation Human Research Review Board. The patients/participants provided their written informed consent to participate in this study.

## Author contributions

MT: manuscript preparation, writing—original draft preparation, and writing—review and editing. CB, SG, CK, CD, and NT-S: writing—review and editing. MB: supervision. All authors have read and agreed to the published version of the manuscript.
