# Supplementary material for: Diné teachings and public health students informing peers and relatives about vaccine education: Providing Diné (Navajo)-centered COVID-19 education materials using student health messengers
Source: Front Public Health. 2022 Dec 14;10:1046634. doi: 10.3389/fpubh.2022.1046634 (PMC9794580; doi:10.3389/fpubh.2022.1046634)
Supplement: Supplementary file 1 [file Table_1.docx]

**Supplementary Table 1. Consensus Panel Results**

|  | Student Feedback | CHR Feedback |
| --- | --- | --- |
| **Language**   - Are the words easy to follow? - Are the appropriate words used to convey the information to a wide range of adults? - Are there any words that need to be replaced or translated? - Is the amount of information appropriate? - Are the explanations easy to follow? - Do you think your family members and friends will be able to relate to the content/wording of these materials? | - Scientific language (mRNA, omicron) may be hard to understand, what language can we use to explain these terms? - Question about “rush the vaccine” is a bit too scientific but it is necessary - Clarifies the myths that were out in the community. - A bit wordy but needs to be addressed - Elderly caretaker who speaks English - would be nice to translate into Navajo - Helpful, gives clear plans for vac vs. unvax - “Post on my fridge” kind of handout | - Would "UNVACCINATED or VACCINATED" be better than " not up to date on your Covid-19 vaccination? - Changing language on vac vs. unvac and making it less confusing - Changing the ‘fact’ to false on the FAQ’s – will help readers understand better |
| **Format and Organization**   - Do you think your family members and friends will find the information presented in these materials relevant, or useful? - Are the explanations and information in these materials easy to follow? Can we present or organize this information in a way that makes it easier to follow? | - Easy to follow, bolding of the words is helpful - Family and friends have been asking about microchip, they would understand - keeping in mind about dad (in 80’s) - Useful - not aware of when to get the 2nd dose and booster. Explains side effects well - Love that its culturally based | - Bullet points; "after 5 full days"; " fever free x 24* hours"; "your symptoms are improving" - Adding borders around the symptoms & tips |
| **Images and Colors**   - Do you think adults will find the images culturally appropriate? - Are the images engaging? - What colors should we use that you think adults would find appealing? - Are there cultural beliefs, values, and practices of the community that we can incorporate into our health education materials? | - Red/maroon pops - Colors are okay, dad has vision problems but could still differentiate - Incorporate native design into handout - Changing color of guys on page 2 to look more native | - Colors look great on all handouts |
| **Content**   - Do the materials provide the information that you feel your family and friends need to know to take appropriate preventive action for the health of their family? - Do the materials answer the questions that you have heard your family and friends raise about the virus and the use of the vaccines? | - Re-infected after getting COVID: stress even then to get the vaccine, “I’ve already had it, and don’t need the vaccine”, language about booster for added protection - Answers most of the questions heard in family - may have more questions but answered the main/common questions. - Incorporation of tips, easy for individuals to follow - Herbal medicine - people are not aware of how to use it when recovering from COVID and after recovering from COVID | - Materials were great, can’t wait to use as CHRs |
