# Supplementary material for: Diné teachings and public health students informing peers and relatives about vaccine education: Providing Diné (Navajo)-centered COVID-19 education materials using student health messengers
Source: Front Public Health. 2022 Dec 14;10:1046634. doi: 10.3389/fpubh.2022.1046634 (PMC9794580; doi:10.3389/fpubh.2022.1046634)

**Supplementary Table 2. COVID-19 Health Education Materials**

COVID-19 Vaccines


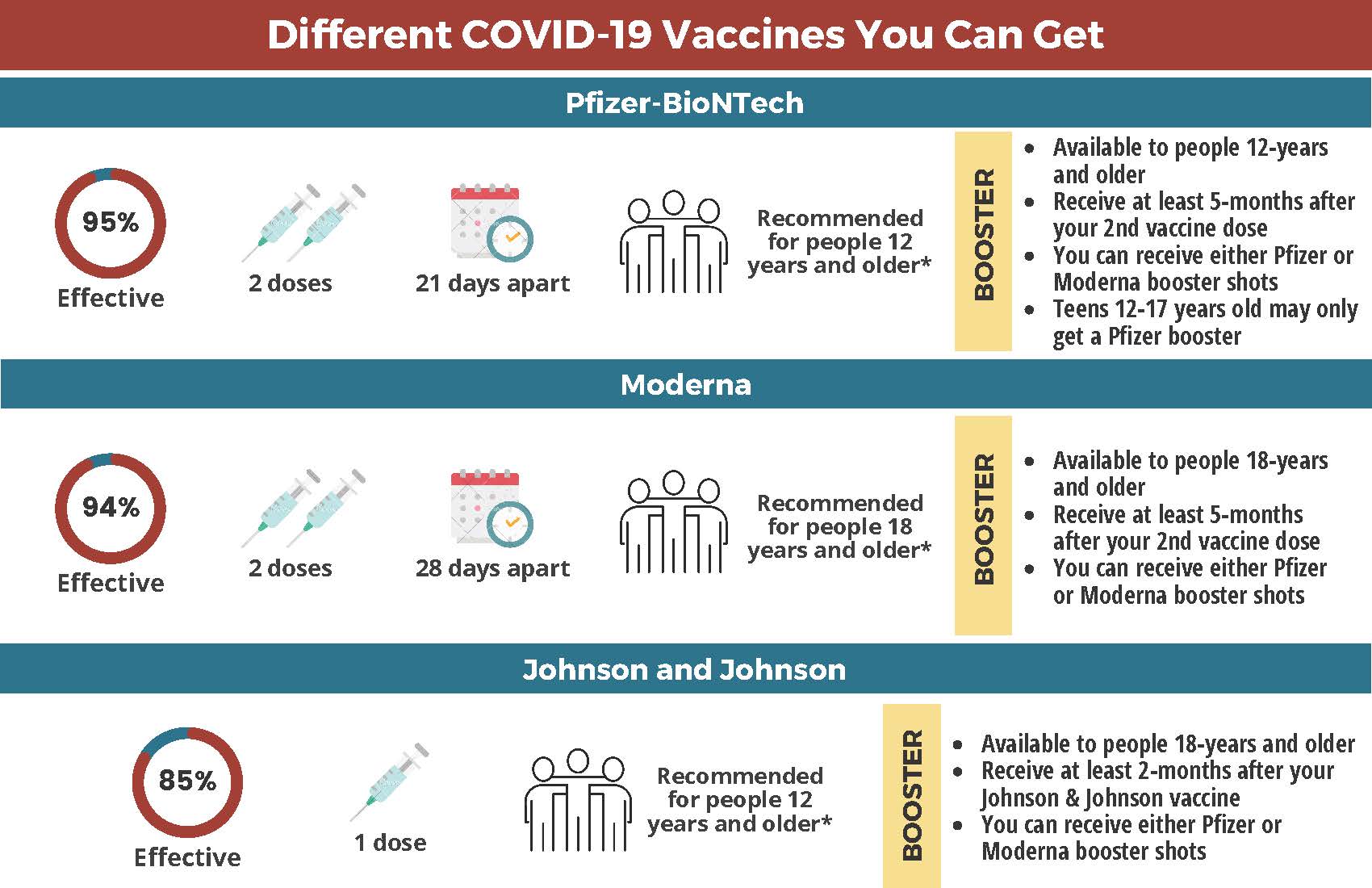


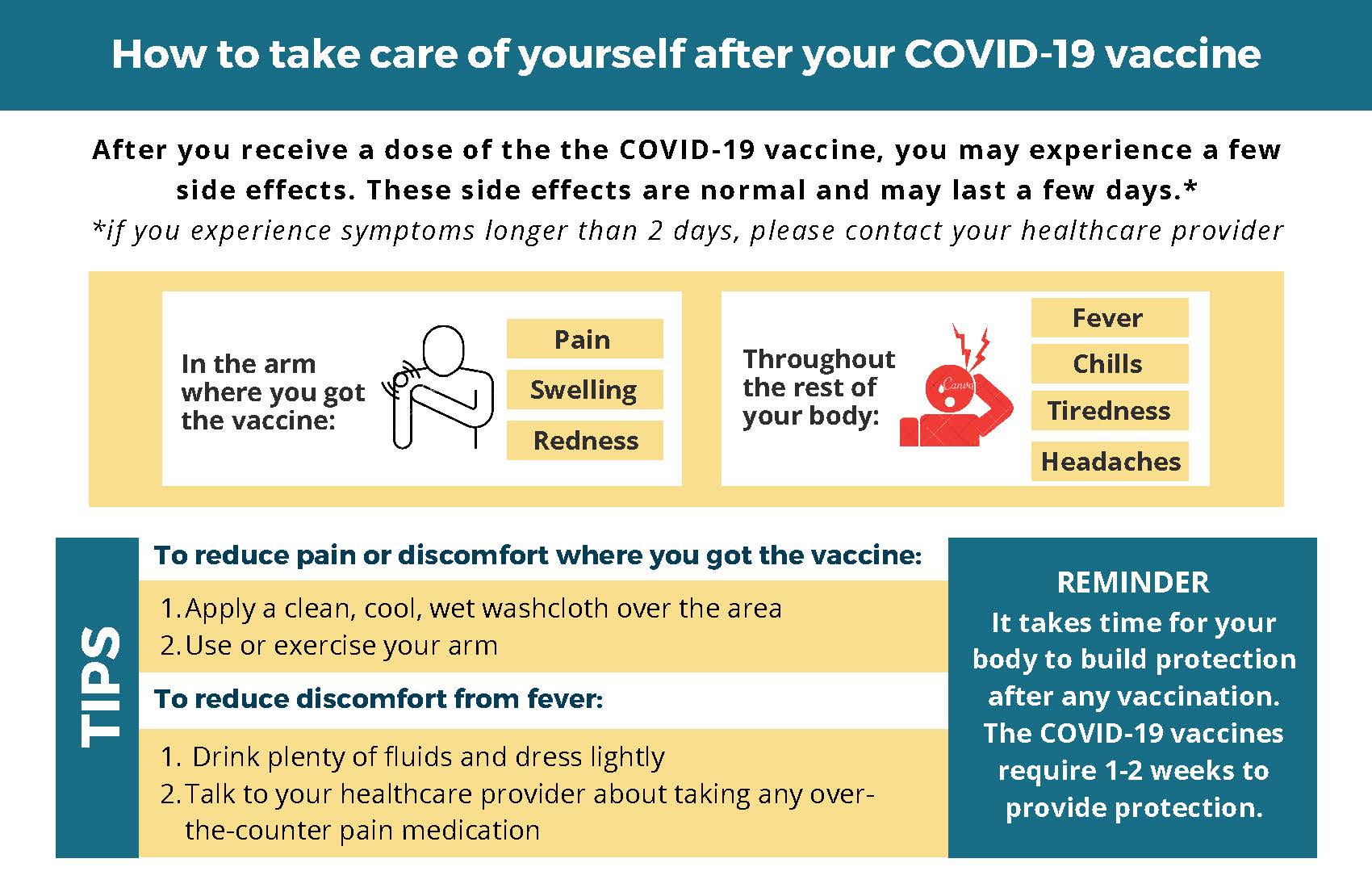


COVID FAQs and Myths


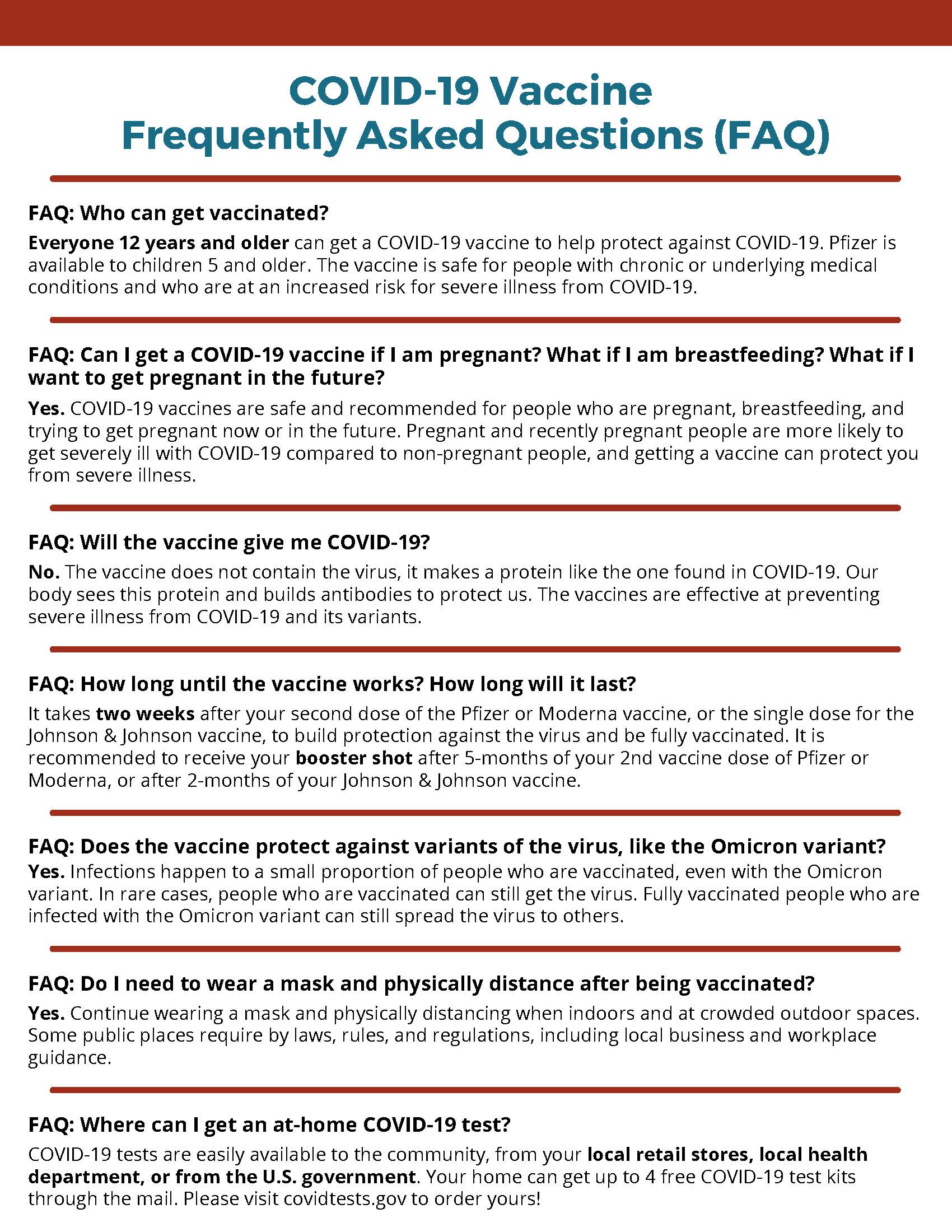


COVID FAQs and Myths (continued)


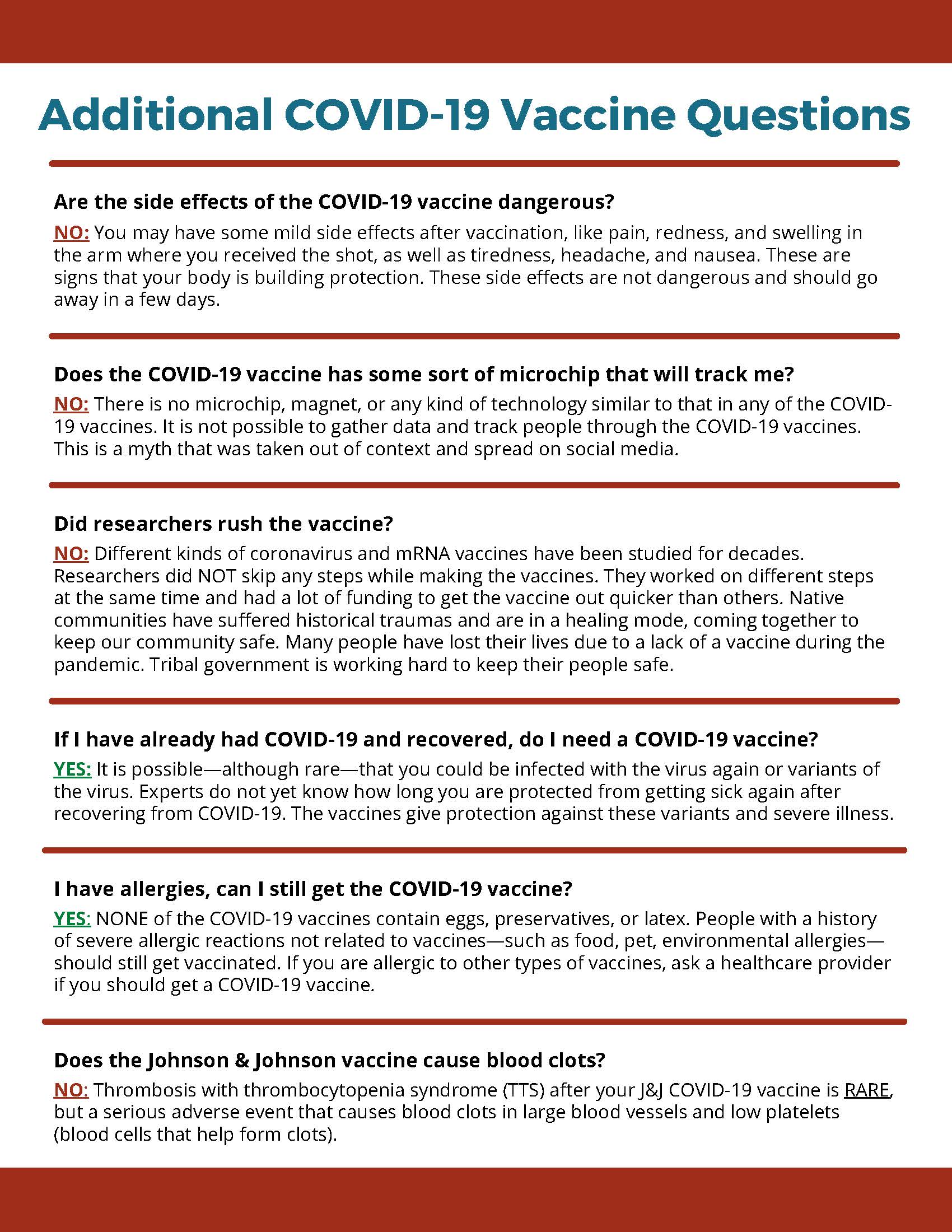


Quarantine vs. Isolation


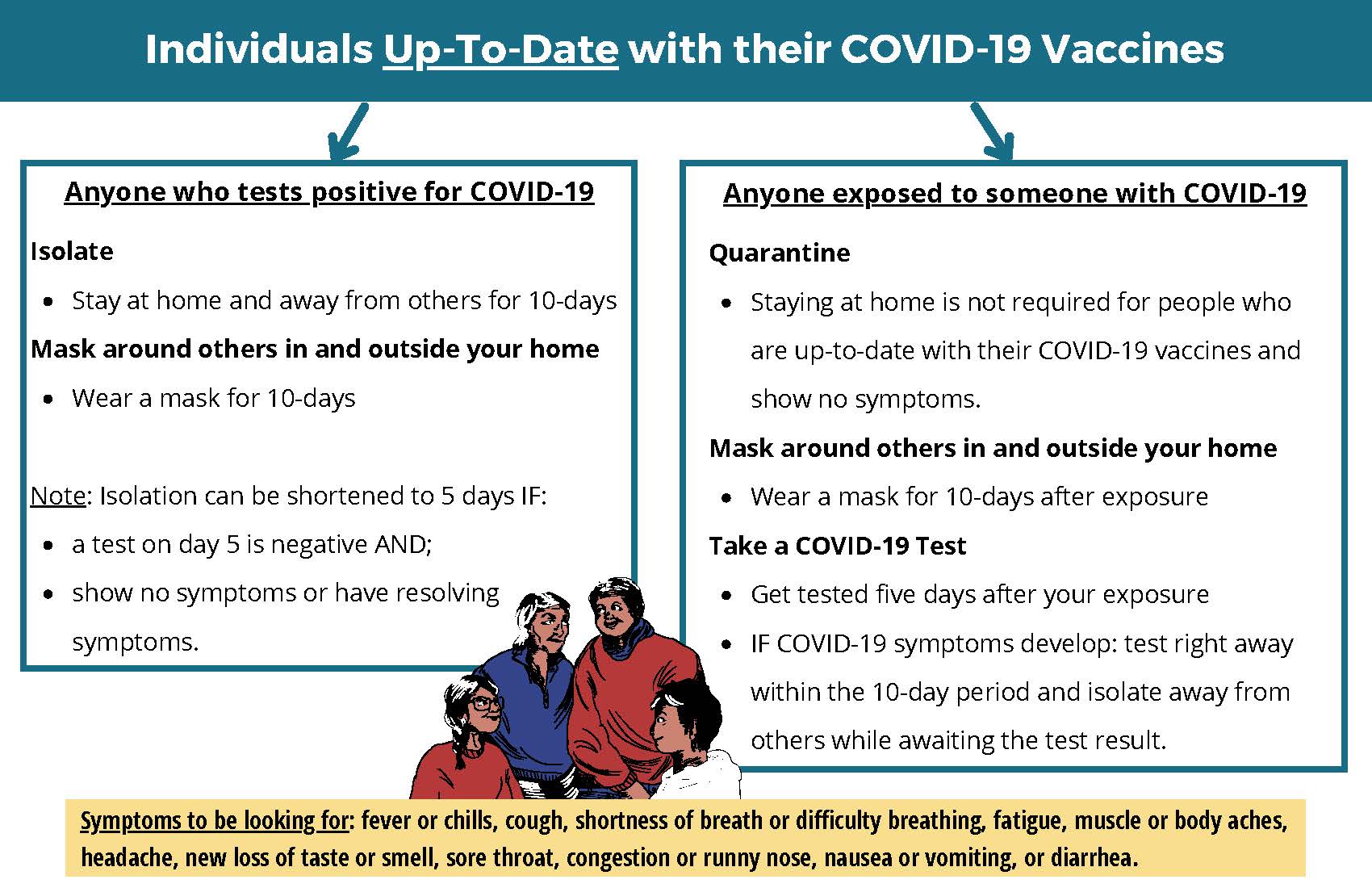

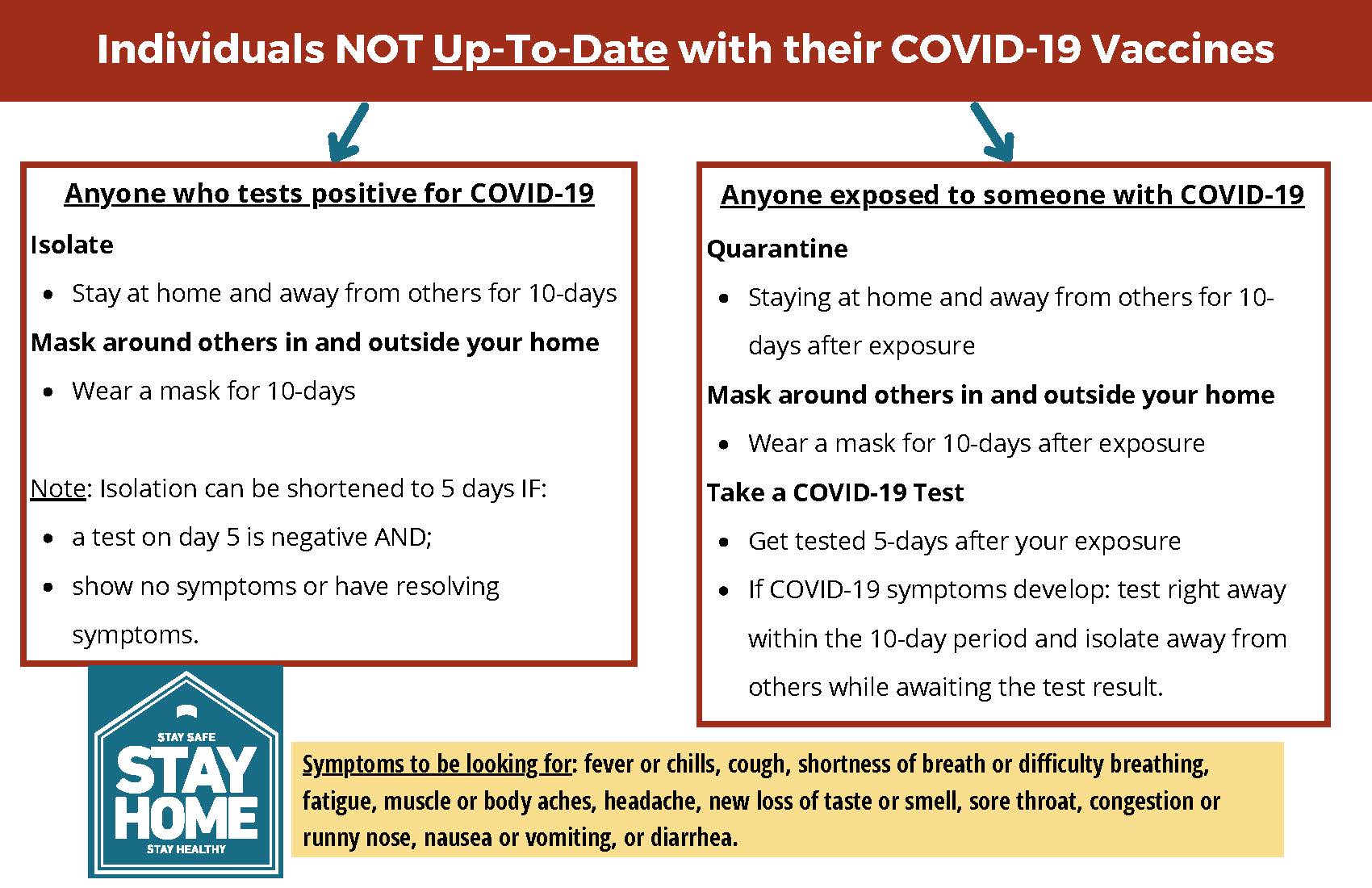


Traditional Knowledge of COVID-19
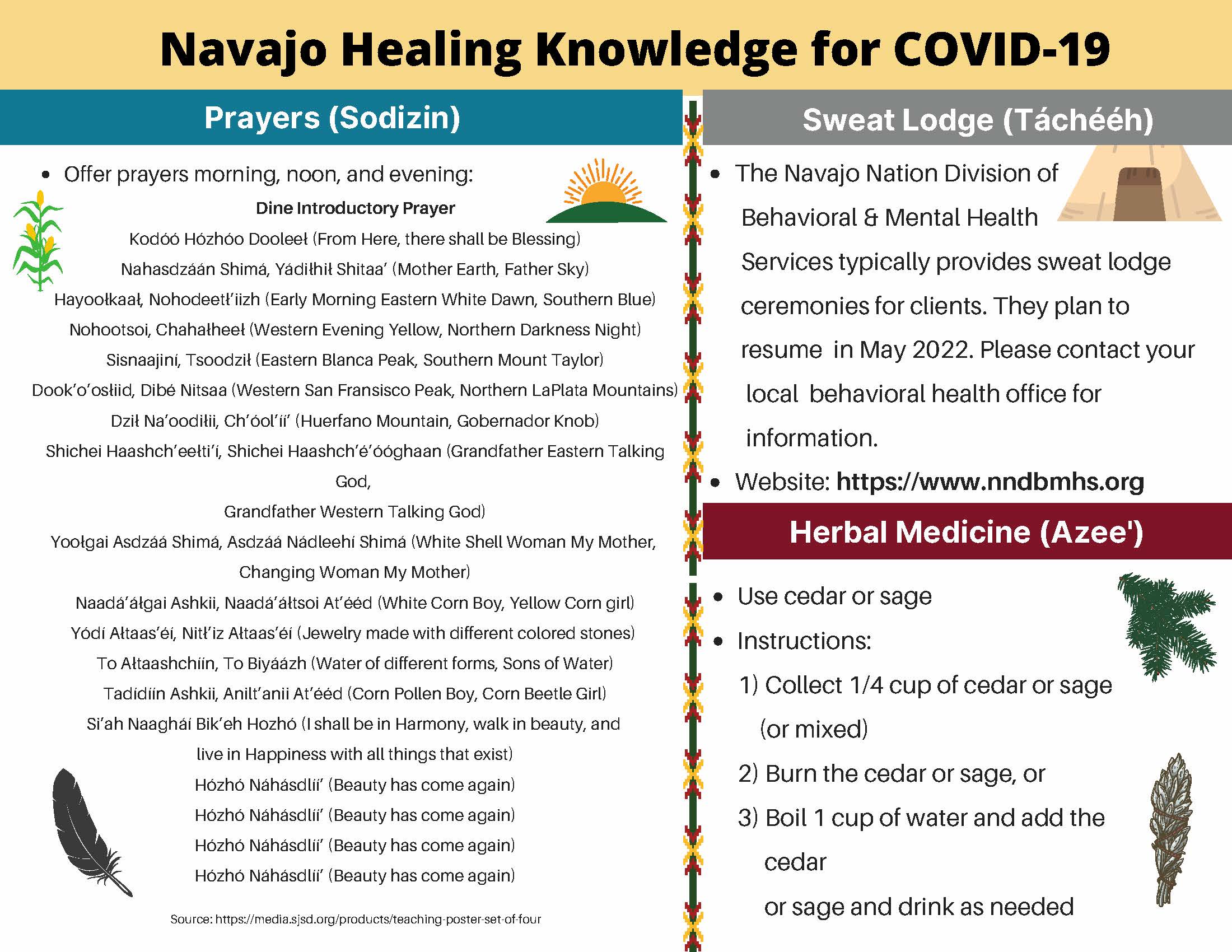

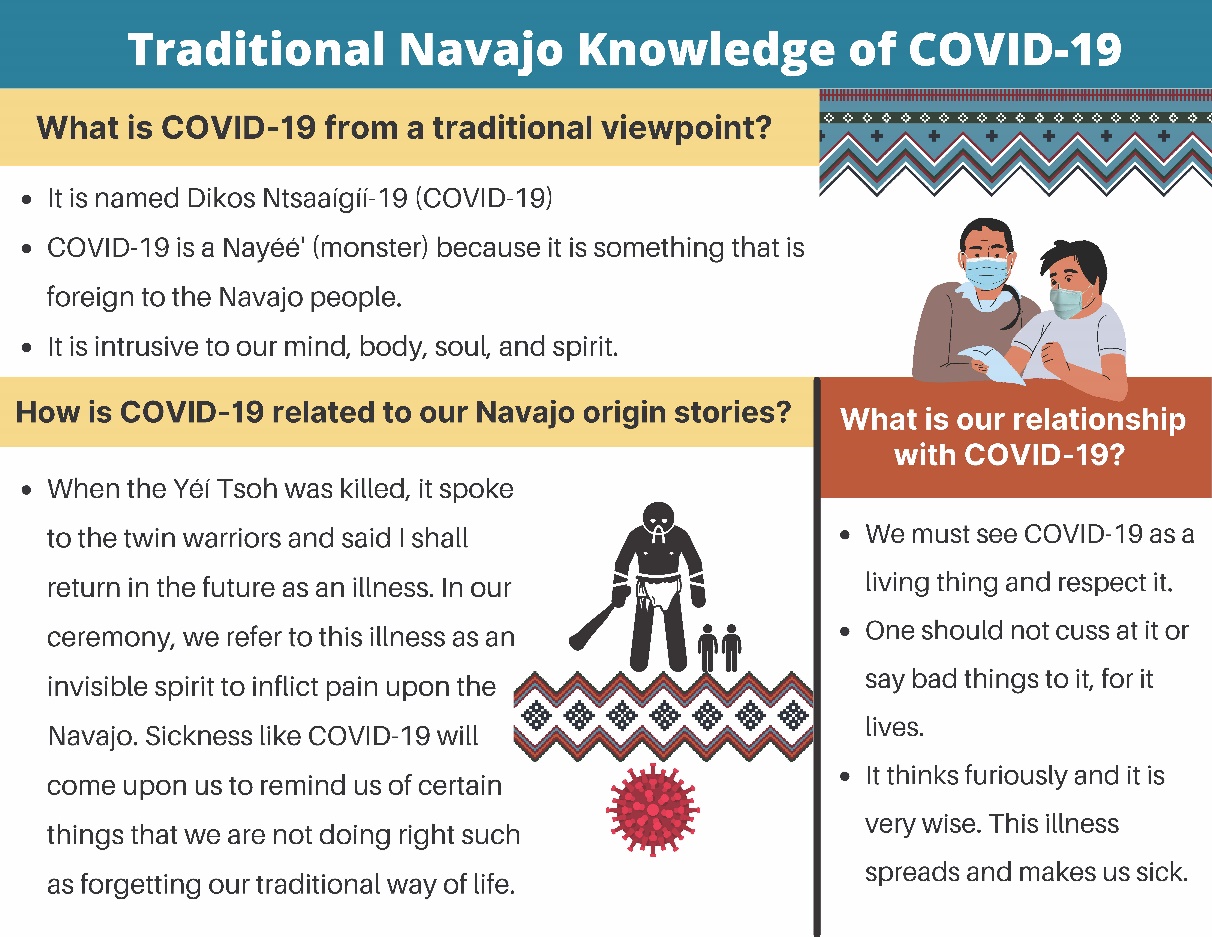


Traditional Knowledge of COVID-19 (continued)


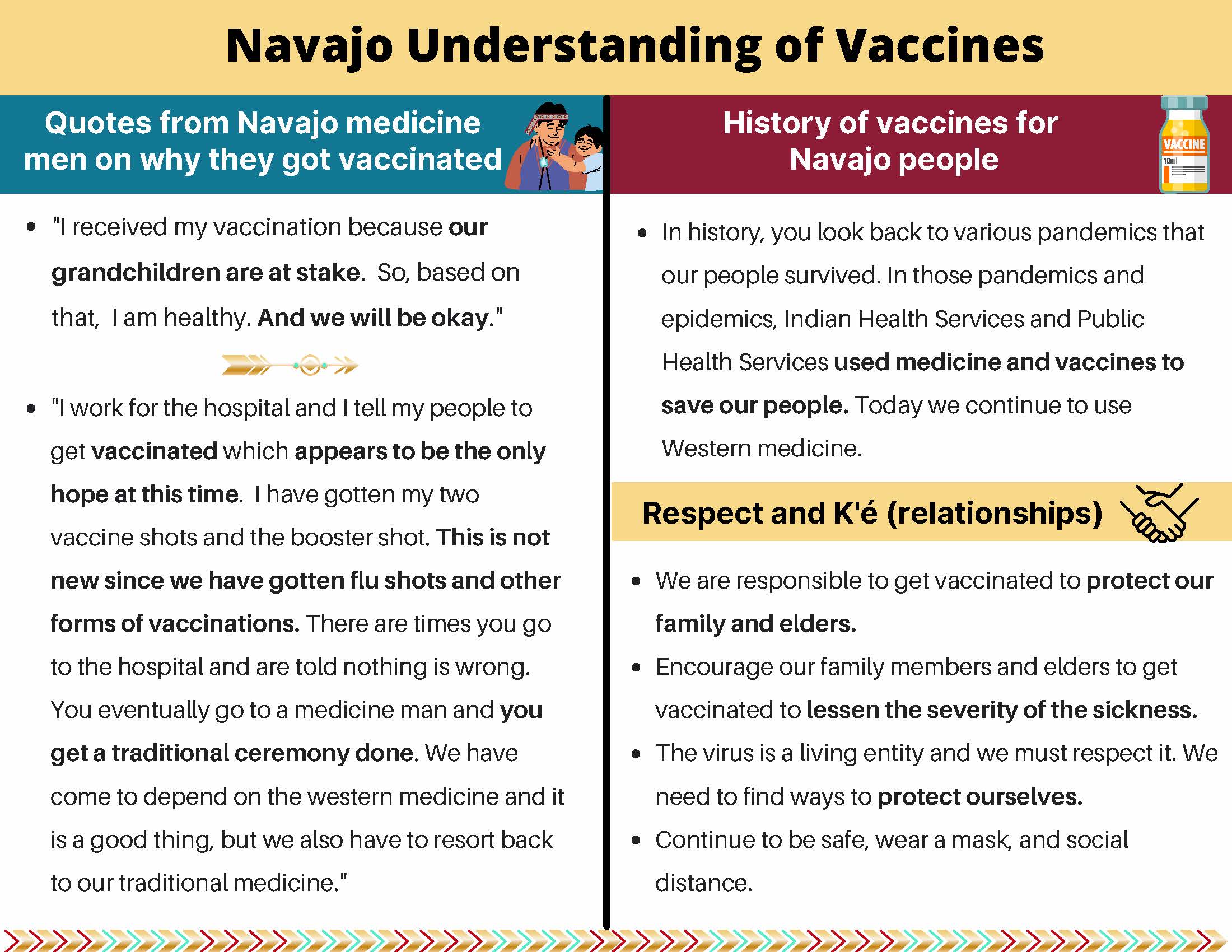

Supplement: Supplementary file 2 [file Table_2.docx]
