# Supplementary material for: Diné teachings and public health students informing peers and relatives about vaccine education: Providing Diné (Navajo)-centered COVID-19 education materials using student health messengers
Source: Front Public Health. 2022 Dec 14;10:1046634. doi: 10.3389/fpubh.2022.1046634 (PMC9794580; doi:10.3389/fpubh.2022.1046634)
Supplement: Supplementary file 3 [file Table_3.docx]

**Supplementary Table 3. Changes in attitudes, perceived behavioral control, subjective norms, and intent to receive vaccine by health messaging recipients**

| **Attitudes** | Pretest  Agreement | Posttest  Agreement | Change | p-value |
| --- | --- | --- | --- | --- |
| Getting vaccinated for COVID-19 is a good idea. | 44.44% | 69.57% | **56.50%** | **0.013** |
| COVID-19 vaccines will work in preventing COVID-19. | 36.36% | 60.47% | **66.30%** | **0.021** |
| If I get the vaccines, I will be less likely to get COVID-19. | 26.67% | 34.09% | 27.80% | 0.298 |
| COVID-19 vaccines protect the health of my community. | 47.83% | 73.33% | **53.30%** | **0.011** |
| Concerns about whether COVID-19 vaccines are safe. | 86.96% | 82.61% | -5.00% | 0.386 |
| Not enough research done on COVID-19 vaccines. | 86.96% | 67.39% | **-22.50%** | **0.023** |
| Concerns about possible side effects of COVID-19 vaccines. | 84.78% | 78.26% | -7.69% | 0.296 |
| Transportation is a barrier (not sure I can get a ride) to a place where I can get the vaccine. | 18.18% | 15.22% | -16.30% | 0.462 |
| Not sure where to go to get the vaccine. | 22.22% | 11.11% | -50.00% | 0.129 |
| **Perceived Behavioral Control** | | | | |
| It will be easy for me to get the vaccines to protect myself from COVID-19. | 60.87% | 76.09% | 25.00% | 0.089 |

| **Subjective Norms** | Pretest  Agreement | Posttest  Agreement | Change | p-value |
| --- | --- | --- | --- | --- |
| Most people who are like me will get vaccinated for COVID-19. | 46.67% | 63.64% | 36.40% | 0.081 |
| Most people who are important to me will get vaccinated for COVID-19. | 69.57% | 84.78% | 21.90% | 0.068 |
| Most people who are important to me think that I should get COVID-19 vaccines. | 73.91% | 84.78% | 14.70% | 0.152 |
| **Intention to Receive COVID-19 Vaccine** | | | | |
| Consider getting COVID-19 vaccines? | 45.65% | 65.22% | **42.90%** | **0.046** |
| Try to get COVID-19 vaccines? | 35.56% | 63.04% | **77.30%** | **0.008** |
| Actually get vaccinated for COVID-19? | 42.22% | 56.52% | 33.90% | 0.124 |
